# Antidepressants for depression after concussion and traumatic brain injury are still best practice

**DOI:** 10.1186/s12888-019-2076-9

**Published:** 2019-03-27

**Authors:** Noah D. Silverberg, William J. Panenka

**Affiliations:** 1 Division of Physical Medicine and Rehabilitation, University of British Columbia, 4255 Laurel St, Vancouver, BC V5Z 2G9 Canada; 2 Rehabilitation Research Program, Vancouver Coastal Health Research Institute, GF Strong Rehab Centre, 4255 Laurel St, Vancouver, British Columbia V5Z 2G9 Canada; 3 Department of Psychiatry, British Columbia Neuropsychiatry Program, University of British Columbia, Vancouver, Canada; 2255 Wesbrook Mall, Vancouver, BC V6T 2A1 Canada; 4 Department of Psychiatry, British Columbia Mental Health and Addictions Research Institute, University of British Columbia, Vancouver, Canada; 2255 Wesbrook Mall, Vancouver, BC V6T 2A1 Canada

## Abstract

**Background:**

Depression is a common complication of traumatic brain injury (TBI). New evidence suggests that antidepressant medication may be no more effective than placebo in this population.

**Main body:**

Selective serotonin reuptake inhibitors are recommended as first-line treatment for depression in contemporary expert consensus clinical practice guidelines for management of TBI. This recommendation is based on multiple prior meta-analyses of clinical trials in depression after TBI as well as depression in the general population. The evidence is mixed. A recent clinical trial and new meta-analysis including that trial found no benefit of antidepressants for depression following TBI. We argue that this finding should not change practice, i.e., patients who present with depression after TBI should still be considered for antidepressant treatment, because they may (1) benefit from robust placebo effects, (2) benefit from an alternative or adjunctive medication if the agent prescribed first does not achieve a depression remission, and (3) make improvements that are not captured well by traditional depression outcome measures, which are confounded by TBI sequelae. Patients with mild TBI are especially appropriate for antidepressant therapy because they, on average, more closely resemble patients with no known TBI history enrolled in typical primary Major Depressive Disorder clinical trials than patients enrolled in TBI trials in placebo-controlled trials published to date.

**Conclusion:**

TBI, and especially *mild* TBI, is not a contraindication for antidepressant therapy. Health providers should routinely screen and initiate treatment for depression after TBI.

## Background

Depression is common after traumatic brain injury (TBI), with at least 1 in 5 patients meeting criteria for a Major Depressive Episode within the first six months [1–3]. This rate is similar across the spectrum of TBI severity. Depression may magnify the burden of physical and cognitive symptoms as well as functional disability after TBI [2, 4], making it an important treatment target.

## Main text

Prior meta-analyses have concluded that antidepressant medications are effective for depression in a variety of neurological disorders [5], including TBI [6]. Correspondingly, selective serotonin reuptake inhibitors are recommended as first-line treatment for depression in recently published expert consensus clinical practice guidelines for management of TBI [7, 8]. However, a recently published systematic review [9] raised doubt about this evidence base. Kreitzer et al. [9] screened 1020 articles published before September 20, 2017 and found 11 eligible pharmacological intervention studies in TBI samples. Their meta-analysis of the five placebo-controlled trials revealed “no benefit of antidepressant over placebo in the treatment of [Major Depressive Disorder] following TBI” (standardized mean difference = − 0.3; 95% confidence interval = − 0.6 to 0.0; I [2] = 17%) [9]. This conclusion might lead some clinicians to not offer antidepressant therapy to their patients who present with depression after TBI, which in our view, would be unfortunate. Even if this meta-analytic finding is “real” (not a Type II error), we argue here that proactive treatment should be considered, especially in *mild* TBI (concussion), for the following reasons.

First, when non-randomized and open-label studies were included in the Kreitzer et al. [9] study as well as prior meta-analyses on the same topic [6, 10], the treatment effect was significant. That is, patients who received antidepressant therapy got better. These gains may be attributable to placebo effects. Even so, placebos may be a powerfully effective treatment for various TBI-related problems [11]. For a patient who presents with depression after TBI, prescribers may have an opportunity to harness placebo effects with on-label use of an active medication.

Second, patients whose depression does not respond to an initial antidepressant trial often benefit from augmentation or switching to an alternative selective serotonin reuptake inhibitor or a selective serotonin-epinephrine reuptake inhibitor. This stepwise approach is recommended in guidelines for TBI management [7]. Placebo-controlled trials such as those synthesized in Kreitzer et al. [9] measure the change in depressive symptoms on the first attempted antidepressant agent in the “average” patient, not the potential for depression remission with stepwise medication trials, as in real-world clinical practice.

Third, traditional depression outcome measures may lose responsiveness when applied in TBI studies, resulting in underestimation of treatment benefit. For example, 3 out of the 5 placebo-controlled trials pooled by Kreitzer et al. [9] measured depressive symptoms with the Hamilton Depression Rating Scale (HAM-D). The authors acknowledge that "instead of demonstrating antidepressants do not work over time, it could be possible that this outcome measure may not change sufficiently with time due to factors related to the underlying TBI [9]. The HAM-D, like most depression measures, includes non-specific symptoms that could reflect structural brain injury. There is external evidence from non-pharmacological treatment trials that the HAM-D and its subscales are suboptimally responsive to improvements in depression after TBI [12].

The rationale for using antidepressants after mild TBI is most compelling. Patients with mild TBI patients may more closely resemble patients with no known TBI history enrolled in typical primary Major Depressive Disorder clinical trials than patients enrolled in TBI trials in placebo-controlled trials published to date. Consider for example the “only study to influence the results of the RCT meta-analysis” in Kreitzer et al’s sensitivity analyses (that is, the only study that, if excluded, would have resulted in a statistically significant meta-analytic finding favoring antidepressants): the recent trial by Fann et al. [13]. These authors only enrolled patients with mild TBI if they had radiological evidence of brain injury and were admitted to a Level 1 trauma center. Many patients with mild TBI do not present to an acute care hospital. Of those who do, a minority will have a trauma-related intracranial abnormality on computed tomography and/or be admitted to hospital. Therefore, the mild TBI patients in Fann et al’s study are not representative of the mild TBI population, but rather reflect a subset at the “severe” end of mild TBI spectrum.

## Conclusions

We argue that TBI, and especially *mild* TBI, is not a contraindication for antidepressant therapy. Proactive detection and treatment of psychiatric problems following TBI of any severity has the potential to improve not only mental health outcomes [14, 15] but also cognition [16–18], somatic symptoms [19, 20], and daily functioning [2, 4, 21]. We recognize that a systematic review of randomized placebo-controlled trials is the highest level of evidence, but caution clinicians against changing their practice on the basis of the Kreitzer et al. [9] study. Clinicians who continue to prescribe will be compliant with practice guidelines [7, 8] and the best available evidence [6, 9, 10]. Selective serotonin reuptake inhibitors (e.g., sertraline or citalopram) are generally recommended as first-line [7, 18, 22, 23]. Efficacy and tolerability of selective serotonin-epinephrine reuptake inhibitors are less well-established [23], but expert consensus guidelines endorse their use [7]. Tricyclic antidepressants are thought to have a less favorable benefit-risk profile, with lowering of the seizure threshold in patients with moderate-severe TBI being a potential concern [8, 23, 24].

Psychotherapy should be offered as an alternative or adjunctive treatment, where accessible [7]. The majority of patients with TBI prefer psychotherapy to pharmacotherapy treatment options [25]. There is insufficient evidence to recommend one modality of psychotherapy over another, but cognitive-behavioural therapies have been most well-studied [23, 26]. Remote delivery, such as by telephone, appears feasible and as effective as in-person therapy [27]. The minimum effective “dose” of psychotherapy is unknown.

Routine screening by rehabilitation and primary care providers can facilitate timely detection of depression after TBI. For example, the Personal Health Questionnaire-9 is a brief self-report depression case-finding tool with strong psychometric properties [12] and diagnostic accuracy in TBI [28]. A positive screening result should trigger a diagnostic clinical assessment with a qualified provider [7, 8].

## Abbreviations

RCT: Randomized controlled trial
TBI: Traumatic brain injury

## References

[1] Ouellet M-C, Beaulieu-Bonneau S, Sirois M-J (2018). Depression in the first year after traumatic brain injury. J Neurotrauma.

[2] Bryant RA, O’Donnell ML, Creamer M, McFarlane AC, Clark CR, Silove D (2010). The psychiatric sequelae of traumatic injury. Am J Psychiatry.

[3] Bombardier CH, Fann JR, Temkin NR, Esselman PC, Barber J, Dikmen SS (2010). Rates of major depressive disorder and clinical outcomes following traumatic brain injury. JAMA..

[4] Zahniser E, Nelson LD, Dikmen S, et al. The temporal relationship of mental health problems and functional limitations following mTBI: a TRACK-TBI study. J Neurotrauma. 2018:neu.2018.6172. 10.1089/neu.2018.6172. 10.1089/neu.2018.6172 PMC6551992 30543138

[5] Price A, Rayner L, Okon-Rocha E (2011). Antidepressants for the treatment of depression in neurological disorders: a systematic review and meta-analysis of randomised controlled trials. J Neurol Neurosurg Psychiatry.

[6] Salter KL, Andrew McClure J, Foley NC, Sequeira K, Teasell RW (2016). Pharmacotherapy for depression posttraumatic brain injury: a meta-analysis. J Head Trauma Rehabil.

[7] Ontario Neurotrauma Foundation (2018). Guidelines for concussion/Mild Traumatic Brain Injury & Persistent Symptoms.

[8] INESS-ONF Clinical practice guideline for the rehabilitation of adults with moderate to severe traumatic brain injury. https://braininjuryguidelines.org/modtosevere/. Accessed 9 Jan 2019.

[9] Kreitzer N, Ancona R, McCullumsmith C, et al. The effect of antidepressants on depression after traumatic brain injury. J Head Trauma Rehabil. 2018:1. 10.1097/HTR.0000000000000439. 10.1097/HTR.0000000000000439 PMC8730802 30169440

[10] Barker-Collo S, Starkey N, Theadom A (2013). Treatment for depression following mild traumatic brain injury in adults: a meta-analysis. Brain Inj.

[11] Polich G, Iaccarino MA, Kaptchuk TJ, Morales-Quezada L, Zafonte R (2018). Placebo effects in traumatic brain injury. J Neurotrauma.

[12] Dyer JR, Williams R, Bombardier CH, Vannoy S, Fann JR (2016). Evaluating the psychometric properties of 3 depression measures in a sample of persons with traumatic brain injury and major depressive disorder. J Head Trauma Rehabil.

[13] Fann JR, Bombardier CH, Temkin N (2017). Sertraline for major depression during the year following traumatic brain injury: a randomized controlled trial. J Head Trauma Rehabil.

[14] Jorge RE, Acion L, Burin DI, Robinson RG. Sertraline for preventing mood disorders following traumatic brain injury a randomized clinical trial. JAMA Psychiatry. 2016;73(10). 10.1001/jamapsychiatry.2016.2189. 10.1001/jamapsychiatry.2016.2189 27626622

[15] Bryant RA, Moulds M, Guthrie R, Nixon RD (2003). Treating acute stress disorder following mild traumatic brain injury. Am J Psychiatry.

[16] Fann JR, Uomoto JM, Katon WJ (2001). Cognitive improvement with treatment of depression following mild traumatic brain injury. Psychosomatics..

[17] Terry DP, Brassil M, Iverson GL, Panenka WJ, Silverberg ND. Effect of depression on cognition after mild traumatic brain injury in adults. Clin Neuropsychol. 2018. 10.1080/13854046.2018.1459853. 10.1080/13854046.2018.1459853 29726314

[18] Silver JM, McAllister TW, Arciniegas DB (2009). Depression and cognitive complaints following mild traumatic brain injury. Am J Psychiatry.

[19] Lange RT, Iverson GL, Rose A. Depression strongly influences postconcussion symptom reporting following mild traumatic brain injury. 2011;26(2):127–137. doi:10.1097/HTR.0b013e3181e4622a. 10.1097/HTR.0b013e3181e4622a 20631632

[20] Fann JR, Uomoto JM, Katon WJ (2000). Sertraline in the treatment of major depression following mild traumatic brain injury. J Neuropsychiatry Clin Neurosci.

[21] Fann JR, Burington B, Leonetti A, Jaffe K, Katon WJ, Thompson RS (2004). Psychiatric illness following traumatic brain injury in an adult health maintenance organization population. Arch Gen Psychiatry.

[22] Warden DL, Gordon B, McAllister TW, et al. Guidelines for the pharmacologic treatment of neurobehavioral sequelae of traumatic brain injury. J Neurotrauma. 2006. 10.1089/neu.2006.23.1468. 10.1089/neu.2006.23.1468 17020483

[23] Fann JR, Hart T, Schomer KG (2009). Treatment for depression following traumatic brain injury: a systematic review. J Neurotrauma.

[24] Wroblewski BA, McColgan K, Smith K, Whyte J, Singer WD. The incidence of seizures during tricyclic antidepressant drug treatment in a brain-injured population. J Clin Psychopharmacol. 1990. 10.1097/00004714-199004000-00009. 10.1097/00004714-199004000-00009 2341586

[25] Fann JR, Jones AL, Dikmen SS, Temkin NR, Esselman PC, Bombardier CH (2009). Depression treatment preferences after traumatic brain injury. J Head Trauma Rehabil.

[26] Stalder-Luthy F, Messerli-Burgy N, Hofer H, Frischknecht E, Znoj H, Barth J (2013). Effect of psychological interventions on depressive symptoms in long-term rehabilitation after an acquired brain injury: a systematic review and meta-analysis. Arch Phys Med Rehabil.

[27] Fann JR, Bombardier CH, Vannoy S (2015). Telephone and in-person cognitive behavioral therapy for major depression after traumatic brain injury: a randomized controlled trial. J Neurotrauma.

[28] Fann JR, Bombardier CH, Dikmen S (2005). Validity of the patient health Questionnaire-9 in assessing depression following traumatic brain injury. J Head Trauma Rehabil.

